# A Video-Based Mobile App as a Health Literacy Tool for Older Adults Living at Home: Protocol for a Utility Study

**DOI:** 10.2196/29675

**Published:** 2022-12-07

**Authors:** Catarina Nunes-Da-Silva, André Victorino, Marta Lemos, Ludmila Porojan, Andreia Costa, Miguel Arriaga, Maria João Gregório, Rute Dinis de Sousa, Ana Maria Rodrigues, Helena Canhão

**Affiliations:** 1 Comprehensive Health Research Center NOVA Medical School|Faculdade de Ciências Médicas, NMS|FCM Universidade Nova de Lisboa Lisbon Portugal; 2 EpiDoC Unit NOVA Medical School|Faculdade de Ciências Médicas, NMS|FCM Universidade Nova de Lisboa Lisbon Portugal; 3 Faculdade de Ciências e Tecnologia Universidade Nova de Lisboa Costa da Caparica Portugal; 4 Unidade de Saúde Pública Algarve II – Barlavento Centro de Saúde de Portimão Portimão Portugal; 5 Direção-Geral da Saúde Lisbon Portugal; 6 Instituto de Saúde Ambiental Faculdade de Medicina Universidade de Lisboa Lisbon Portugal; 7 Escola Superior de Enfermagem de Lisboa Lisbon Portugal; 8 Catolica Research Centre for Psychological, Family and Social Wellbeing Lisbon Portugal; 9 Faculdade de Ciências da Nutrição e Alimentação Universidade do Porto Porto Portugal; 10 Programa Nacional para a Promoção da Alimentação Saudável Direção-Geral da Saúde Lisbon Portugal; 11 Episaúde – Associação Científica Évora Portugal

**Keywords:** mobile app, technology, treatment adherence, health literacy, seniors, older adults

## Abstract

**Background:**

People aged ≥65 years are more likely to have health problems related to aging, polypharmacy, and low treatment adherence. Moreover, health literacy levels decrease with increasing age.

**Objective:**

The aim of this study is to assess an app’s utility in promoting health-related knowledge in people aged ≥65 years.

**Methods:**

We developed a simple, intuitive, and video-based app (DigiAdherence) that presents a recipe, nutritional counseling, and content on physical activity, cognitive exercise, motivation to adhere to treatment, fall prevention, and health literacy. A convenience sample of 25 older adults attending the Personalized Health Care Unit of Portimão or the Family Health Unit of Portas do Arade (ACeS Algarve II – Barlavento, ARS Algarve, Portugal) will be recruited. Subjects must be aged ≥65 years, own a smartphone or tablet, be willing to participate, and consent to participate. Those who do not know how to use or do not have a smartphone/tablet will be excluded. Likewise, people with major cognitive or physical impairment as well as those living in a long-term care center will not be included in this study. Participants will have access to the app for 4 weeks and will be evaluated at 3 different timepoints (V0, before they start using the app; V1, after using it for 30 days; and V2, 60 days after stopping using it). After using the app for 30 days, using a 7-point Likert scale, participants will be asked to score the mobile tool’s utility in encouraging them to take their medications correctly, improving quality of life, increasing their health-related knowledge, and preventing falls. They will also be asked to assess the app’s ease of use and visual esthetics, their motivation to use the app, and their satisfaction with the app. Subjects will be assessed in a clinical interview with a semistructured questionnaire, including questions regarding user experience, satisfaction, the utility of the app, quality of life (EQ-5D-3L instrument), and treatment adherence (Morisky scale). The proportion of participants who considered the app useful for their health at V1 and V2 will be analyzed. Regarding quality of life and treatment adherence perceptions, comparisons will be made between V0 and V1, using the *t* test for dependent samples. The same comparisons will be made between V0 and V2.

**Results:**

This study was funded in December 2019 and authorized by the Executive Board of ACeS Algarve II – Barlavento and by the Ethics Committee of NOVA Medical School (99/2019/CEFCM, June 2020). This protocol was also approved by the Ethics Committee for Health (16/2020, September 2020) and the Executive Board (December 2020) of the Regional Health Administration of the Algarve, IP (Instituto Público). Recruitment was completed in June 2021.

**Conclusions:**

Since the next generation of older adults may have higher digital literacy, information and communication technologies could potentially be used to deliver health-related content to improve lifestyles among older adults.

**International Registered Report Identifier (IRRID):**

PRR1-10.2196/29675

## Introduction

Following the trend observed in other developed countries, Portugal’s population is aging rapidly, and Eurostat estimates it will be the oldest country in the European Union by 2050 [1]. Although medical and scientific progress has made it possible to increase the average life expectancy, this rapid aging of the population is associated with an increasing number of people with multiple chronic diseases and disabilities.

In the 2015 National Health Examination Survey, 3.9 million Portuguese people (57.8%) reported having at least one chronic condition. The occurrence of chronic disease was more frequent in people aged 65-74 years and in those with a lower educational level [2]. Four years later, the tendency remained. According to Portugal: Country Health Profile 2019, 53% of people ≥65 years of age have at least one chronic disease. Nonetheless, many reported having two or more chronic illnesses [3].

Additionally, data from the Epidemiology of Chronic Diseases (EpiDoC) cohort, a prospective cohort study based on a representative sample of the Portuguese population, showed that there is a high prevalence of multimorbidity among older adults and that the frequency of coexisting multiple diseases increases with age (72.8% in people aged 65-69 years vs 83.4% in people ≥80 years). Importantly, this was associated with unhealthy lifestyle behaviors, such as physical inactivity [4]. Multimorbidity and polypharmacy, which are of particular concern in an aging population, are also associated with poor treatment adherence [5-7].

The literature suggests that low health literacy levels are associated with worse health outcomes. For instance, those with lower health literacy tend to resort to emergency services more frequently, have higher health expenditures, and do not make as much use of preventive medicine services [8-10].

Income, age, race, and education level are considered contributing factors to health literacy levels. Authors have reported that even minor cognitive decline in older nonimpaired adults is associated with health literacy decline [11].

Because of the current COVID-19–related need for social distancing, it is essential to implement new nonpharmacological strategies to support older adults living at home by promoting their physical, mental, and nutritional health and increasing their treatment adherence and health-related knowledge. Digital patient-centered solutions can potentially help us create these much-needed alternatives. Studies have reported several apps for older adults to promote social interaction, health, and well-being [12-15]. An example is STARFISH, a smartphone-based app that monitors users’ physical activity by counting their daily steps and facilitates social support [12]. In terms of treatment adherence, evidence suggests it can be improved with apps designed for older adults, even if they have no smartphone or tablet experience [16].

We have previously developed an informative and motivational, home-based, 12-week program (Saúde.Come) aimed at promoting healthy eating at a low cost and the practice of regular physical exercise. The program was delivered by an interactive television application [17]. The pilot study showed Saúde.Come was well accepted, reduced food insecurity, and improved the participants’ physical function [18].

Several projects are being developed under the umbrella of the Portuguese Health Literacy Action Plan 2019-2021 [19], which aims to continuously, consciously, and sustainably improve the health literacy level of those living in Portugal. The Portuguese Directorate-General for Health plans to disseminate, free of charge, an educational mobile app to promote physical activity, mental health, and nutritional health in older adults. Given our experience [17], we have joined them in the effort and developed such an app (DigiAdherence). In this study, we aim to assess the DigiAdherence mobile app’s utility in promoting health literacy.

## Methods

### The DigiAdherence App

We have developed a simple, intuitive, and video-based mobile app using the Android Studio software and the following programming languages:

- Java for operations, such as user interaction when clicking a button, and the various video-linked options including pause, fast-forward, rewind, and full-screen mode
- XML for the system design implementation and each of the app’s actions
- Gradle for Java development and implementation, enabling the app’s adaptation to different types of Android systems/mobile phones

Our user interface was developed with a reader-friendly mindset (straightforward content coupled with eye-catching, simple, and consistent design). Color and contrast were used for optimal visibility (#02796B for the foreground and #FFFFFF for the background; contrast ratio of 5.31:1). Videos are stored locally within the app, allowing it to function on demand and without connecting to the internet. To build the executable file (APK), we used Android Studio’s “Build APK” option.

The aging process is associated with the development of various chronic noncommunicable diseases (like high blood pressure or rheumatic diseases) and the decline of one’s senses, motor function, and cognition. Altogether, these may compromise a person’s ability to perform certain physical and mental tasks, making them vulnerable to life-threatening events such as falls. We have previously shown there is a high prevalence of chronic diseases and unhealthy lifestyle behaviors among the Portuguese older adult population, highlighting the need for dedicated interventions [4]. The DigiAdherence app integrates 6 short videos designed to motivate older adults, encourage the consumption of healthy food, improve the practice of physical activity, prevent falls, encourage cognitive exercise, and increase treatment adherence. The app’s contents were developed by a multidisciplinary team of professionals, including 1 chef, 1 nutritionist, 1 personal trainer, 1 psychologist, and 2 rheumatologists. Video length varies between 2 and 8 minutes. In essence, the DigiAdherence app is intended to be an extension of the comprehensive care provided by a person’s primary care health care professional.

**Figure 1:**
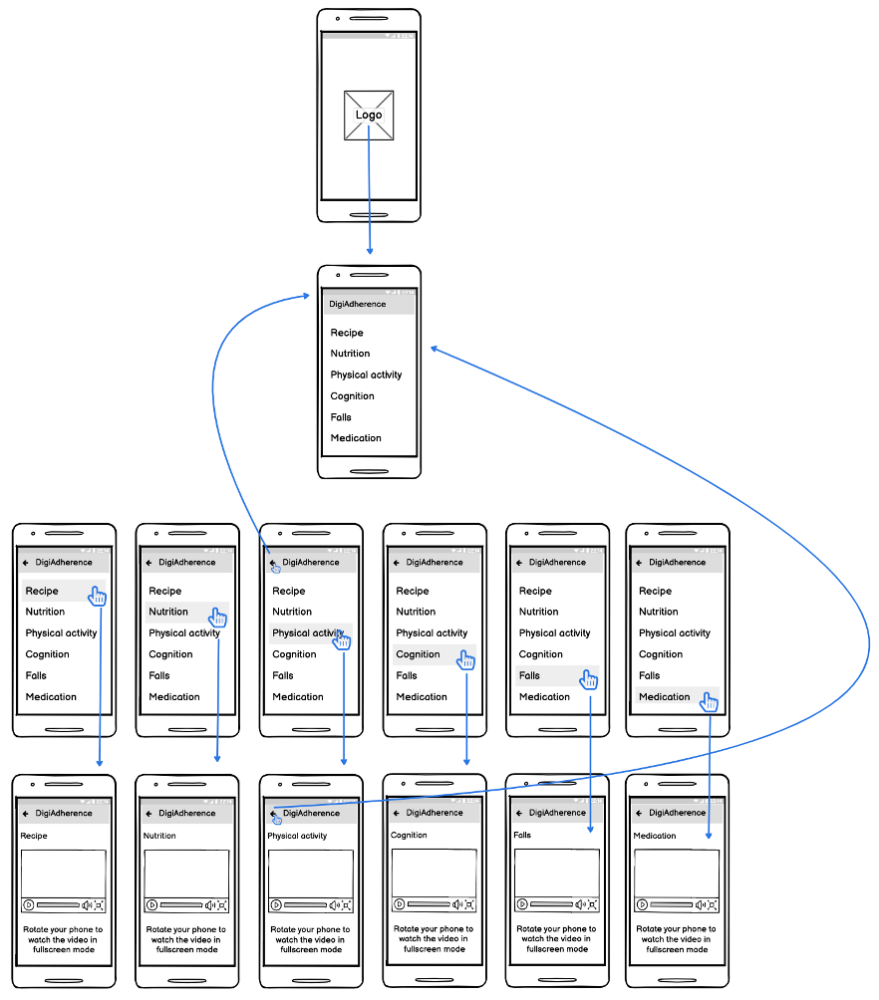
DigiAdherence app wireflow.

The app can be accessed using an Android smartphone or tablet. DigiAdherence’s launch screen appears instantly when the app starts up and is quickly replaced with the app's 6-option main menu, with each menu button leading to a distinct health-related thematic video (wireflow depicted in Figure 1). Half of the video content was used in a previous successful study conducted by our research group [17,18]. In the first section, a chef teaches the users how to make a healthy carrot soup; in option number 2, a personal trainer demonstrates a series of physical activity exercises that older adults can do while sitting in a chair; in section number 3, a nutritionist talks about sugar replacement options; in option number 4, a psychologist talks about the importance of doing cognitive exercises, giving some examples of the types of exercises that older adults can do; in section number 5, a rheumatologist lists a series of techniques that can be adopted by older adults to prevent falls on the street or at home; finally, in section 6, a rheumatologist talks about the risk of polypharmacy and gives tips on what older adults should do to ensure they take their medication as prescribed. Note that every professional made sure to speak clearly, without using medical or technical jargon, keeping their messages simple and easy to understand. Full-screen mode is enabled by rotating the smartphone or tablet horizontally.

DigiAdherence is an offline app that users can access multiple times and at any time. Even though there are no notifications sent by the app, we believe the participants will access its contents when, for instance, they feel the need to exercise without leaving the house or want to learn more about nutrition. The app was designed to get users to see a health-related video of their choice with just 2 taps.

As an offline app, we will not be quantifying the app’s engagement metrics such as frequency of use, exit rate, or repeat usage. Our focus will be on the app’s content utility in improving self-reported health-related knowledge (7-point Likert scale). For more details on the variables this study will assess, please read the Data Collection subsection.

### Study Description

#### Participants and Recruitment

**Figure 2:**
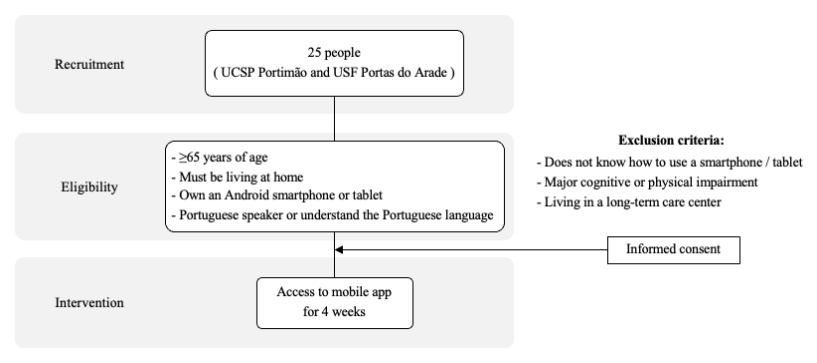
DigiAdherence study design overview. UCSP: Personalized Health Care Unit; USF: Family Health Unit.

This study will include a convenience sample of 25 patients, aged 65 years or older, who attend the Barlavento Health Center Cluster (ACeS), Regional Health Administration (ARS) of the Algarve, Portugal (Figure 2). The selection of potential participants will be carried out by their primary health care physician. Subjects’ recruitment will occur at the Personalized Health Care Unit of Portimão and at the Family Health Unit Portas do Arade, both belonging to ACeS Algarve II – Barlavento, Portugal. Those who show an interest in participating in the study will be contacted by a Public Health physician who will invite them to an in-person visit at the Public Health Unit of ACeS Algarve II – Barlavento. At this visit, the physician will explain the study to the older adult and, if he/she agrees to participate and meets the study’s inclusion criteria, ask him/her to sign the informed consent. The recruitment phase is expected to last 2 months.

To be eligible for participation in this pilot study, inclusion criteria are as follows. Participants (male/female) must (1) be aged ≥65 years, (2) be living at home, (3) own an Android smartphone or tablet, (4) be a Portuguese speaker or understand the Portuguese language, (5) visit the Personalized Health Care Unit of Portimão or the Family Health Unit of Portas do Arade (ACeS Algarve II – Barlavento, ARS Algarve, Portugal), and (6) be willing and consent to participate.

Inclusion criteria were not limited to a specific set of chronic diseases or medications to increase the odds of older adults who are smartphone owners joining our pilot study.

Exclusion criteria include the following: (1) individuals who do not understand Portuguese, (2) individuals who do not know how to use or do not have an Android smartphone/tablet, (3) individuals who have a major cognitive or physical impairment and/or (4) individuals who are living in a long-term care center.

#### Study Design and Procedures

After being recruited and consenting to their participation, subjects will have to attend 3 scheduled visits at their primary health care center. All clinical evaluation and data collection visits will be carried out at the Public Health Unit of ACeS Algarve II – Barlavento, Portugal.

At the first visit (V0), we will collect information about the participant’s health status, medication, health literacy, quality of life, and frequency of falls (if subjects fell in the last month, how many times did they fall? Answer options: 1, 2, 3, 4, 5, >5). After participants install the app on their smartphone or tablet, they will be given a demonstration on how the app works and have the chance to clarify any questions that may arise regarding the app’s functioning. The health care professional performing the participants’ recruitment will also encourage them to use the app. Access to the DigiAdherence app will be granted for 4 weeks. There will be no notifications or triggers.

After a month, there will be a second visit (V1) to reassess the subjects’ health status, adherence to technology, quality of life, frequency of falls, and health literacy. The third visit (V2) will occur 2 months after the end of the exposure to the app. The participants’ health status, quality of life, frequency of falls, and health literacy will be reassessed in this consultation.

This study does not comprise an app usability testing, since DigiAdherence’s content was adapted from the Saúde.Come project [17,18], a multidisciplinary 12-week, home-based program focused on improving dietary and physical activity through an interactive television app. Saúde.Come has been fully implemented and tested in a similar population and shown to be feasible and acceptable for use by users [18]. Furthermore, DigiAdherence is an offline app with no interaction or dynamic elements, consisting mainly of educational content for which no difficulties in engaging with the app were anticipated.

#### Data Collection

As previously described, this study entails three evaluation moments: the initial one (V0; before the participant starts using the app), after using the app for 30 days (V1), and 60 days after stopping using DigiAdherence (V2) (Table 1). In terms of variable selection, we selected knowledge as the target determinant since it is one of the main factors contributing to the adoption of self-management practices, according to various theories of behavior change such as the Capability, Opportunity, Motivation, Behavior Model [20,21].

**Table 1:** Variables to be assessed within the DigiAdherence study.

	Baseline	Visit 1	Visit 2
Visit number	V0	V1	V2
Timing (weeks)	0	4	12
Informed consent	X		
Inclusion/exclusion criteria	X		
Participant identifier	X		
Birth date	X		
Sex	X		
Weight and height	X	X	X
Educational level	X		
Concomitant medications	X	X	X
Self-reported chronic diseases	X	X	X
Self-reported number of falls in the last month	X	X	X
Self-reported health literacy	X	X	X
Health-related quality of life (EQ-5D-3L instrument; score from –1 to 1)	X	X	X
Treatment adherence (8-item Morisky Medication Adherence Scale; score from 0-8; <6 denotes low adherers, 6 to <8 denotes medium adherers, and 8 denotes high adherers)	X	X	X
Self-reported adherence to the technology, including an evaluation of the app’s instrumental and noninstrumental attributes on a 7-point Likert scale (1=most negative response, 7=most positive response); attributes include utility in the correct uptake of medication, utility in improving quality of life, utility in improving health-related knowledge, utility in preventing falls, ease of use, visual esthetics, motivation to use the app, and satisfaction with the app		X	

At V1 and using a 7-point Likert scale, older adults will be asked to score the mobile tool’s utility in taking their medications correctly, improving quality of life, increasing their health-related knowledge, and preventing falls.

Using the same scoring system, participants will also be asked to assess the app’s ease of use and visual esthetics, as well as their motivation to use the app and satisfaction.

All assessments will be based on a structured questionnaire. All visits will focus on treatment adherence (Morisky scale [22]) and the prevention of falls. We will also assess perceptions regarding quality of life (assessed by the Portuguese version of the EQ-5D-3L questionnaire [23], the European Quality of Life questionnaire with 5 dimensions and 3 levels) and health-related knowledge (participants will be asked if they felt that the app’s content increased their health-related knowledge and if they made use of the information delivered to prevent falls and to correctly take their medication). At these visits, sociodemographic data (age, gender, level of education) and clinical history will also be collected, including concomitant medication, comorbidities, and history of falls.

#### Statistical Analysis

Statistical analysis will be performed using the STATA software (version 16; StataCorp LLC). Participants will be characterized in terms of sociodemographic variables, health, quality of life, and fall risk. Normally distributed continuous variables will be described as mean and standard deviation, while nonnormal distributed ones will be presented as median and interquartile range. Categorical variables will be reported as frequencies or proportions.

We will analyze the proportion of participants who considered the app useful for their health in V1 and V2. Regarding quality of life (EQ-5D-3L [23]) and self-reported medication uptake (Morisky scale [22]), comparisons will be made between V0 and V1, using the *t* test for dependent samples if data are normally distributed; if not, Wilcoxon signed-rank test will be used. The same comparisons will be made between V0 and V2. Statistical significance will be considered when *P*<.05.

#### Ethical Issues

This study was submitted and authorized by the Executive Board of ACeS Algarve II – Barlavento and by the Ethics Committee of NOVA Medical School (99/2019/CEFCM, June 2020), NOVA University of Lisbon, Portugal. This protocol was also submitted to and approved by the Ethics Committee for Health (16/2020, September 2020) and the Executive Board of the Regional Health Administration of the Algarve (December 2020), IP (Instituto Público). All procedures will follow the principles of Good Clinical Practice and the Declaration of Helsinki (Fortaleza revision, 2013). Participants will be included in the study solely after obtaining their informed consent.

Although this pilot study is based on the use of a mobile phone app, the latter is an educational app that will only deliver short videos with a recipe, nutritional counselling, physical activity content, cognitive exercises, health literacy content, and motivation to adhere to treatment content. It should be noted that this technology will not collect or transmit, through communication networks, any personal data related to the participants’ health. Thus, according to the General Regulation on Data Protection; Regulation (EU) 2016/679 of the European Parliament and the Council of April 27, 2016; and Regulation No. 1/2018, this study is exempt from notification to the Portuguese Data Protection Authority.

## Results

Recruitment was completed in June 2021. Data analyses are ongoing. Research findings will be made available to communities of interest through peer-reviewed journals and scientific conferences.

## Discussion

### Overview

This study aims to assess the DigiAdherence app’s utility in improving health-related knowledge among older adults living at home. Based on our experience with information and communication technologies, we anticipate that the accurate educational content of the DigiAdherence app will prove useful for users of the app and contribute to the improvement of their health-related knowledge and encourage them to exercise and eat healthy.

Although older adults can sometimes become overwhelmed by the ever-changing world of technology, the next generation of older adults will be fairly digitally literate. Because people aged 65 years and over often have more health problems related to aging, polypharmacy, and low treatment adherence, information and communication technologies could potentially be used to deliver health-related content to improve lifestyles.

Those with lower health literacy are more receptive to video-based education [24,25]. Our research group has previously implemented a 12-week, home- and video-based intervention program (Saúde.Come) to reduce food insecurity in older adult populations using an interactive television app [17]. Saúde.Come was found to be feasible and well accepted by its users [18]. Moreover, a study [26] showed that 97% of community-dwelling older adults use television as their primary source of health information.

The acceptability of video across different age groups (ie, including older adults) and the exponential adoption of digital technology makes video an attractive communication tool to conveniently deliver health information to older adults no matter where they are.

### Limitations

To measure medication uptake, we chose an instrument (Morisky Scale) that only quantifies self-reported oral medication uptake. Hence, injectable medication adherence will not be measured, constituting a study limitation. It should also be taken into consideration that the overall impact of the intervention on participants’ health-related knowledge may be diminished, considering the app’s short-form content and the lack of dynamic elements like triggers or notifications.

### Conclusions

Since the next generation of older adults is somewhat tech-savvy, short mobile videos could potentially be used to deliver accurate health-related knowledge to improve lifestyles among older adults.

## Abbreviations

ACeS: Health Center Cluster
ARS: Regional Health Administration
EQ-5D-3L: European Quality of Life questionnaire with 5 dimensions and 3 levels
